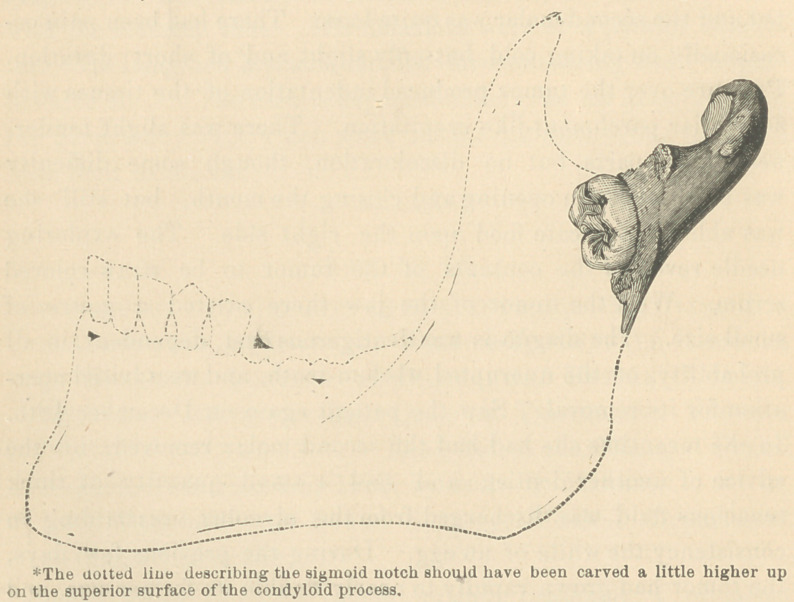# Dentigerous Cyst

**Published:** 1884-01

**Authors:** John S. Marshall

**Affiliations:** Late Instructor of Dental Surgery, Medical Department Syracuse University


### ARTICLE II.

**DENTIGEROUS CYST LOCATED IN THE INFERIOR MAXILLA, DEPENDENT UPON AN INVERTED WISDOM-TOOTH, WITH REMOVAL OF NECROSED PORTION OF RAMUS, AND ENTIRE CONDYLE. REPRODUCTION OF THE LOST OSSEOUS TISSUE AND PERFECT MOVEMENT OF THE JOINT.** Read before the Chicago Dental Society, November 6th, 1883, by JOHN S. MARSHALL, M. D., Late Instructor of Dental Surgery, Medical Department Syracuse University.

GENTLEMEN:—At the request of our President, I promised to prepare an essay for this evening's discussion, but from the limited time at my disposal, I have been unable to do so; rather, however, than disappoint you I will relate a case that came under my observation while in Syracuse, N. Y., in consultation with Prof. John Van Duyn, of that city, at whose suggestion and approval I have prepared the case for publication.

Anna T., aged 16 years, light complexion, well nourished and in general good health, consulted me November 21, 1881, at the request of Prof. John Van Duyn, for a swelling of the left inferior maxilla situated in the neighborhood of the molar teeth. The history is as follows :

In the autumn of 1880, the young lady first noticed a slight external swelling over the left inferior maxilla opposite the first molar tooth and just above the base of the jaw. In August, 1881 the swelling having increased in size, and the first molar, which was badly decayed, having become quite loose, she consulted a dentist, who pronounced it a case of alveolar abscess, and extracted the tooth. When the case first came under my observation, the tumor of the jaw was about the size of a hen's egg, and the swelling extended to the maxillary articulation and in front of the ramus. The wisdom-tooth of that side was not erupted and the second molar was quite loose. There had been pain occasionally on taking cold, but only slight and of short duration. Pressure over the tumor produced indentation of the tissues with a peculiar parchment-like crepitation. There was slight tenderness of the parts, but no discoloration, though some difficulty was experienced in opening and closing the mouth, but still she was able to masticate food upon the right side. The exploring needle revealed the contents of the tumor to be straw-colored serum. With the tumor of the jaw there existed a goitre of small size. The diagnosis was dentigerous cyst, dependent, in all probability, on the unerupted wisdom-tooth, and we advised operation for its removal. Saw the patient again on December 18th. In the meantime she had had the second molar removed, by the advice of another dentist, and said a small quantity of thick tenacious fluid was discharged from the alveolus, resembling in consistency the white of an egg. During the previous few days, the tumor had grown rapidly to nearly double its former size, and extended from the site of the first molar backward and upward to the maxillary articulation. December 19, 1881, operated upon the patient, at her home, for the removal of the offending tooth. She was laid upon a table with the shoulders and head slightly elevated, so as to get a clear view of the parts, and placed under the influence of ether. An incision was then made through the tissue in a longitudinal direction with the jaw, from the angle to the second bicuspid tooth upon the summit of the gums, and a transverse incision in the region of the second molar.

The contents of the cyst were then removed, which consisted of a thick dark yellow serum, slightly mixed with pus; in quantity at least four ounces. On passing the finger into the cyst several sharp spiculæ of bone could be felt upon the sides and bottom of the cavity,—probably the remains of the alveoli of the extracted teeth,—and in the posterior part of the cavity well up towards the sigmoid notch and at the base of the condyloid process, the wisdom-tooth could be distinctly felt; it was in an inverted position, the grinding surface directed downward, forward, and outward, and incomplete in development,—the crown only being formed. (See cut.*) This was easily dislodged with an elevator, and extracted by the aid of the bullet forceps.

*The dotted line describing the sigmoid notch should have been carved a little higher up on the superior surface of the condyloid process.

It was now revealed by a further examination that the condyloid process and the posterior part of the ramus were separated from the coronoid process, and the anterior portion, and detached from the surrounding tissues, the periosteum having been entirely separated from this portion of the bone; this was removed through the cyst and incision in the mouth. The extracted condyle showed evidences of necrosis, and upon examination it was further discovered that the cyst had extended so far upwards and backwards as to nearly sever the condyle from the coronoid process and body of the jaw, and the force applied to dislodge the tooth, though very moderate, no doubt completed it. The condyle was also separated from its fibro-cartilage.

Although the lower part of the ramus, posterior part of the body, and the entire condyloid process were lost, it was deemed best to crush the lateral walls of the cyst, which were so thin that they were not capable of giving any measure of support to the remaining portions of the jaw. None but favorable symptoms followed the operation, regular visits being discontinued December 28, 1881. The wound entirely closed at the end of a few weeks, the discharge having been very small in amount. August 20, 1883, I had the opportunity of seeing the young lady, and found a fully formed ramus and articulation with very little deformity—only a slight flattening of that side was observable, in the region of the angle and ramus of the jaw. The mobility of the joint and the functions of the jaw are perfect and the patient not conscious of any defect. The favorable results of the case were largely dependent, no doubt, upon the periosteum having been left almost entire and the absence of extensive suppuration, as evidenced by the fact that but little pus was found in the cyst.

Malgaigne was the first to lay down the rule that as far as possible the periosteum should be preserved in resections, and out of this has grown all the operations of conservative surgery known as osteoplasty.

Osseous tissue is, however, sometimes reproduced from the soft parts surrounding the bone, as demonstrated by the researches of such pathologists as Duhamel, Müller, Sömmering, Heine, Seymour, Wagner, Flourens, and others.

The remarkable features of this case are the position of the wisdom-tooth, viz., at the sigmoid notch; the death of the condyle, in all probability from the presence of the fluid, and the seemingly almost perfect reproduction of the lost ramus and condyle, together with the restoration of the mobility of the articulation and functions of the jaw. Reproduction of bone is more frequent in the lower maxilla than in the other bones of the face, but the regeneration of so large a portion, including the temporo-maxillary articulation, is very rare.

Garretson mentioned the case of a little German boy six years of age, upon whom he operated for the removal of a necrosed full half of the lower jaw, the result of an attack of measles, which, at the end of two years, was so completely reproduced as to give natural motions of the jaw, and to leave a scarcely to be observed deformity.

Heath cites also a single case, that of a man 22 years of age, who came under his care for the removal, for necrosis following typhoid fever, of the whole body of the lower jaw, including about a third of the right ramus and the condyle. The operation was performed March 3, 1869, when the body of the jaw was removed from the right angle to the second bicuspid tooth of the left side. June 16, a second operation was required to remove a portion of dead bone from the right ramus, and again on October 30, a third operation was demanded, and the entire right condyle, with about a third of the ramus, was removed. Mr. Heath says, "Perhaps the most singular feature in the case is the fact that the man has now (December) as perfect movement of the jaw as if no disease had existed, notwithstanding that at the last operation, the whole of the right condyle was removed entire, with about a third of the ramus. The repair has, in fact, been as complete as possible." So far as I have been able to ascertain, very few cases are on record in which so large a portion of the jaw and condyle were reproduced, with perfect mobility of the joint.

## Figures and Tables

**Figure f1:**